# Postoperative Refractive Prediction Error Measured by Optical and Acoustic Biometry after Phacovitrectomy for Rhegmatogenous Retinal Detachment without Macular Involvement

**DOI:** 10.1155/2019/5964127

**Published:** 2019-05-02

**Authors:** Masashi Sakamoto, Izumi Yoshida, Takahiro Sodeno, Asao Sakai, Hidetaka Masahara, Takatoshi Maeno

**Affiliations:** Department of Ophthalmology, Toho University Sakura Medical Center, Sakura-shi, Japan

## Abstract

**Introduction:**

The aim of this study was to investigate the postoperative prediction error measured by optical biometry and acoustic biometry in eyes after phacovitrectomy for rhegmatogenous retinal detachment (RRD) with no macular involvement.

**Methods:**

Forty-nine eyes of 49 patients (32 male, 17 female; mean age 62.6 ± 7.5 years) with RRD without macular involvement who underwent phacovitrectomy (RRD group) and 49 eyes of 33 patients (21 male, 12 female; mean age 74.1 ± 7.1 years) without macular disease who underwent cataract surgery (control group) were included in this retrospective comparative study. The difference between the preoperative predictive value and the postoperative refractive value was measured both by optical and acoustic biometry and compared in each group.

**Results:**

The postoperative refractive error calculated by acoustic biometry was −0.81 ± 0.75*D* and that calculated by optic biometry was −0.44 ± 0.77*D* in the RRD group. The postoperative refractive error calculated by acoustic biometry was −0.21 ± 0.64*D* and that calculated by optic biometry was 0.27 ± 0.71*D* in the control group. Significant myopic shifts were observed in the RRD group using both acoustic biometry and optic biometry but not in the control group.

**Conclusion:**

Phacovitrectomy for RRD with no macular involvement resulted in a significant myopic shift when compared with cataract surgery alone in patients without macular disease when calculated by both acoustic biometry and optic biometry.

## 1. Introduction

Rhegmatogenous retinal detachment (RRD) and cataract are often operated on simultaneously because development of cataract after vitrectomy is common [1]. Moreover, pars plana vitrectomy for RRD is more difficult to perform in phakic eyes [2–5]. Therefore, in Europe and Asia, it is common to perform phacovitrectomy for RRD [3–5]. However, a myopic shift after phacovitrectomy has been reported in patients with vitreomacular disorders, such as epiretinal membrane (ERM), macular hole (MH), and RRD [6–10].

Myopic shift has been observed when the macula is not attached in eyes with RRD because of errors in the measurement of axial length [10]. However, myopic shift of refractive error has also been reported after phacovitrectomy in eyes with ERM and in those with MH, in which the measurement of axial length is not considered to be difficult [6, 7, 9, 11]. Gas tamponade during anterior intraocular lens (IOL) replacement has been identified as a cause of myopic shift in eyes with MH [9]. Axial length measured by optic biometry is not accurate in patients with poor fixation because of deviation in the visual axis [12]. Because RRD without macular involvement should have a good fixation, axial length of those cases can be measured accurately.

There are no reports of myopic shift in eyes with RRD and no macular involvement when measurements are obtained by optic biometry and acoustic biometry. In this study, we investigated the prediction error in eyes with RRD without macular involvement measured by both acoustic biometry and optic biometry.

## 2. Materials and Methods

This retrospective, observational comparative study included 49 eyes of 49 patients (32 males, 17 females; mean age 62.6 ± 7.5 years) with RRD and no macular involvement who underwent phacovitrectomy (RRD group) and 49 eyes of 33 patients (21 males, 12 females; mean age 74.1 ± 7.1 years) without macular disease who underwent cataract surgery (control group) at the Toho University Sakura Medical Center between April 2015 and May 2018.

The study protocol was approved by the ethics committee at the Toho University Sakura Medical Center (approval number S18083) and adhered to the tenets of the Declaration of Helsinki. Written informed consent was obtained from all subjects after the study design and risks/benefits of participation were explained via the Toho University Sakura Medical Center website in accordance with the guidelines for clinical research set out by the Japanese Ministry of Health, Labour and Welfare.

Patients who underwent phacovitrectomy with segmental scleral buckling and encircling and those whose IOL was fixed on the anterior capsule because of rupture of the posterior capsule were excluded. Patients in whom measurements could not be obtained by both acoustic biometry and optic biometry and whose axial length was >26 mm or <22 mm were also excluded, as were those who had undergone intraocular surgery or had a history of trauma.

Axial length was measured by both acoustic biometry ultrasonography (UD-6000; Tomey Corporation, Nagoya, Japan) and optic biometry (IOLMaster; Carl Zeiss, Oberkochen, Germany, and Jena, Germany). The examinations included manifest refraction and autokeratometry (K) using an RK-F1 keratometer (Canon Inc., Kanagawa, Japan).

Anterior chamber depth (ACD) was measured using optic biometry. RRD without macular involvement was confirmed by fundus examination and optical coherence tomography. Postoperative refractive outcomes were compared with the predicted refractive value measured by both optic biometry and acoustic biometry. Postoperative refraction was measured at least 1 month after vitrectomy.

### 2.1. Cataract Surgery

The surgical procedure was performed under sub-Tenon local anesthesia using 2% lidocaine. All patients underwent phacoemulsification and aspiration (via a 2.8 mm clear corneal incision at the superior limbus) and posterior capsular implantation of an acrylic foldable IOL.

### 2.2. RRD Surgery

The surgical procedure was performed under retrobulbar local anesthesia using a combination of 2% lidocaine and 0.75% ropivacaine. All patients underwent phacoemulsification and aspiration (via a 2.8 mm corneoscleral incision) and posterior capsular IOL implantation with an acrylic foldable IOL. A standard pars plana vitrectomy was performed using a 25-gauge system (Constellation; Alcon, Inc., Fort Worth, TX) in all cases. Sclerotomies were placed 3.5 mm from the limbus. Core vitrectomy was performed, and, if not already present, a posterior vitreous detachment was induced after staining the vitreous cortex with triamcinolone acetonide. The posterior vitreous detachment was induced to the vitreous base as far as possible, followed by vitreous shaving with scleral indentation. Any untreated retinal holes or tears and lattice degeneration were treated by endolaser photocoagulation followed by gas tamponade using 20% SF_6_.

X-70 (Santen, Osaka, Japan; A-constant: 118.9 for ultrasonography and 119.4 for the IOLMaster) was used in 24 eyes (49.0%) with RRD and in 4 eyes (8.2%) with no macular disease. XY-1 (Hoya, Tokyo, Japan; A-constant: 118.9 for ultrasonography and 119.2 for the IOLMaster) was used in 24 eyes (49.0%) with RRD. AN6K (Kowa, Tokyo, Japan; A-constant: 118.9 for ultrasonography and 119.0 for the IOLMaster) was used in one eye (2.0%) with RRD. SN60WF (Alcon, Inc., Fort Worth, TX, USA; A-constant: 118.7 for ultrasonography and 119.0 for the IOLMaster) was used in 45 eyes (91.8%) without macular disease. The SRK/T formula was used to calculate the IOL power [13].

### 2.3. Statistical Analysis

All statistical analyses were performed using Statcel software (OMS Publishing Inc., Saitama, Japan). Student's *t*-test was used for between-group comparison of the refractive error. The Mann–Whitney *U* test was used to compare between-group differences in the axial length, ACD, and K-average. The chi-squared test was used to investigate any sex-related differences. A *P* value <0.05 was considered statistically significant.

## 3. Results

The mean axial length in eyes with RRD was 24.32 ± 0.90 mm when measured by optic biometry and 24.05 ± 0.89 mm when measured by acoustic biometry; the difference was statistically significant (*P* < 0.01, Mann–Whitney *U* test). The mean axial length in the control group was 23.65 ± 0.86 mm when measured by optic biometry and 23.36 ± 0.85 mm when measured by acoustic biometry; this difference was also significant (*P* < 0.01, Mann–Whitney *U* test; Table 1).

The mean postoperative refractive error was −0.81 ± 0.75*D* measured by acoustic biometry and −0.44 ± 0.77*D* by optic biometry in the RRD group and −0.21 ± 0.64*D* by acoustic biometry and 0.27 ± 0.71*D* by optic biometry, respectively, in the control group (Table 2).

Using axial measurements, a significantly greater myopic shift was recorded by acoustic biometry than by optical biometry in the RRD group (*P* < 0.05, paired *t*-test; Table 3). Similarly, significantly greater myopic shifts were recorded by acoustic biometry than by optical biometry in the control group (*P* < 0.05, paired *t*-test; Table 3). Significant myopic shifts were observed in the RRD group using acoustic biometry and optic biometry when compared with the control group (*P* < 0.05, Student's *t*-test; Table 4).

## 4. Discussion

Refraction after cataract surgery is presently predicted by partial coherence laser interferometry and ultrasonographic acoustic biometry. Optic biometry is a noncontact device that takes measurements down to the retinal pigment epithelium layer and uses a fixation beam that assists with measurements along the visual axis, whereas the axial length obtained by acoustic biometry is from the signal of the internal limiting membrane. Optic biometry is reportedly more accurate than acoustic biometry in eyes with cataract [14].

Recently, in Europe and Asia, phacovitrectomy has been performed in several types of vitreoretinal disease because it is common for cataracts to develop after vitrectomy, and pars plana vitrectomy is more difficult to perform in a phakic eye [1–5]. In general, phacovitrectomy has been performed in patients who are expected to have a good visual prognosis; thus, precise evaluation of the refractive error is a critical issue.

There have been several reports of myopic shift after vitrectomy from measurements recorded by using optic biometry and acoustic biometry [6–10]. Kovacs et al. suggested that myopic shift results from underestimation of the axial length on acoustic biometry ultrasonography because of the thicker macula in eyes with ERM [6]. In contrast, Manvikar et al. [8] reported that there was no tendency for a myopic shift in IOL power estimation using optic biometry, while Falkner-Radler et al. [7] reported that optic biometry detected a myopic change after combined phacovitrectomy despite the IOL power calculation. Higher myopic shifts after vitrectomy with gas tamponade have been reported in eyes with MH than in eyes with ERM [9]. However, in eyes with poor visual acuity, the axial length cannot be measured accurately because of poor fixation, which leads to deviation of the visual axis [12]. Therefore, we compared the axial length measured in eyes with RRD, no macular involvement, and good fixation with that measured in eyes with good visual acuity.

The measurements of myopic shift in the RRD group obtained by optic biometry were more accurate than those obtained by acoustic biometry. A measurement error of 100 *μ*m results in a postoperative error of 0.28*D*^14^. In this study, the mean axial length in the eyes with RRD was 24.32 ± 0.90 mm when measured by optic biometry and 24.05 ± 0.89 mm when measured by acoustic biometry. The difference in axial length measured by acoustic biometry and optic biometry leads to a myopic shift of −0.5*D* for acoustic biometry when compared with optic biometry in the RRD group. This difference in axial length measurement could reflect the fact that optic biometry measures back to the retinal pigment epithelium layer, whereas acoustic biometry measures back to the internal limiting membrane.

Furthermore, the refractive value tended to indicate a greater myopic shift calculated by acoustic biometry because acoustic biometry generally detects an area of 0.3 mm^2^, which is larger than the area of 0.05 mm^2^ detected by optic biometry, and acoustic biometry may measure a different location in the macula [15]. This might lead to the estimated axial length being shorter than the actual axial length calculated by acoustic biometry relative to optic biometry. Therefore, prediction error is more likely when measurements are obtained by acoustic biometry than when they are obtained by optic biometry.

In our study, myopic shifts were detected by both optic biometry and acoustic biometry in eyes with RRD, which is consistent with a previous report [10]. However, in eyes without macular disease, the myopic shifts were detected only by acoustic biometry, which has also been reported previously [14]. Measurements obtained by optic biometry were more accurate than those obtained by acoustic biometry even in eyes with RRD, and no macular involvement that might be considered to have a small deviation in the visual axis; we detected myopic shifts in these eyes by both acoustic biometry and optic biometry. These findings indicate that the myopic shifts were caused by phacovitrectomy. Previous reports have attributed this myopic shift to replacement of vitreous with aqueous fluid, which results in a slight decrease in the refractive value after vitrectomy [16–18]. The same studies have also reported that vitreous is replaced by aqueous fluid after vitrectomy and that the refractive value decreases slightly, thereby resulting in a myopic shift [16–18]. Theoretically, the myopic shift in vitrectomized eyes could be −0.5*D*^18^.

Alternatively, previous studies have suggested anterior displacement of the IOL by the complete gas fill achieved after phacovitrectomy as a potential mechanism of myopic shift [7, 19, 20]. Vitrectomy for RRD usually includes gas tamponade. A further study is needed to investigate the difference in ACD before and after vitrectomy.

In the present study, the myopic shift in the eyes of the RRD group was −0.6*D* to −0.7*D* as measured by both optic biometry and acoustic biometry and was greater than that associated with cataract surgery alone. The reason for this myopic shift was possibly due to the replacement of vitreous with aqueous fluid and the anterior displacement of the IOL. Further research is needed to investigate the difference in the refractive error before and after vitrectomy without gas tamponade in eyes without macular disease, such as those with simple vitreous hemorrhage.

This study has several limitations. One limitation is that the axial length of the eyes in the RRD group was longer than that in the control group. A longer axial length has been reported as a cause of postoperative prediction error [11]. Further studies in which axial length is matched to that in the control group are needed. Studies of IOL positioning have reported that the IOL moves slightly forward during the first postoperative week but that this shift is neutralized by a slight backward movement within 3 months [21]. Therefore, our follow-up duration of 1 month may have been too short, and a smaller refractive error may be seen during the longer-termfollow-up. Further studies that include a longer duration of follow-up are needed. Another shortcoming of this study is that we did not examine the difference in the K value between before and after vitrectomy. Falkner-Radler et al. speculated that the myopic shift in their combined surgery group calculated by optic biometry might result from the postoperative K2 value being significantly higher than the baseline K2 value [7]. A further study is needed to investigate the difference in the K value between before and after vitrectomy. Finally, the preoperative error in axial length measurement of 0.3 mm results in a 0.75*D* difference in the IOL power calculation, which is clinically significant [22]. Finally, axial length after vitrectomy was not measured in our study. Again, further research is required to determine the change in axial length after vitrectomy, given that several studies have suggested that myopic shift might reflect a postoperative increase in axial length caused by scleral thinning or stretching around the sclerotomy site after vitrectomy [7, 10, 23, 24].

## 5. Conclusions

Phacovitrectomy for RRD without macular involvement resulted in a significantly greater myopic shift on both acoustic biometry and optic biometry than that after cataract surgery alone in patients without macular disease. Measurements obtained using optic biometry were more accurate than those obtained using acoustic biometry in eyes with RRD and no macular involvement.

## Figures and Tables

**Table 1:** Patient demographic and clinical characteristics.

	RRD group	Control group	*P* value
Males	32	21	
Females	17	12	0.548
Age (years)	62.6 ± 7.5 (48–82)	74.1 ± 7.1 (57–85)	*P* < 0.001
** **Axial length (mm), optical biometry	24.32 ± 0.90 (22.76–25.99)	23.65 ± 0.86 (22.26–25.77)	0.00026
** **Axial length (mm), acoustic biometry	24.05 ± 0.89 (22.58–25.75)	23.36 ± 0.85 (22.03–25.77)	0.000114
K-average (Dpt)	43.67 ± 1.36 (41.19–47.38)	44.48 ± 1.37 (41.54–47.05)	0.002276
ACD (mm)	3.42 ± 0.33 (2.57–4.09)	3.03 ± 0.42 (2.26–4.08)	*P* < 0.001

*Note*. Data are expressed as mean ± standard deviation and range. The proportion of male patients was analyzed by the chi-squared test. The values for age, axial length, K-average, and ACD were analyzed by the Mann–Whitney *U* test. ACD, anterior chamber depth; RRD, rhegmatogenous retinal detachment.

**Table 2:** Comparison of refractive prediction error between the RRD group and the control group.

	Actual postoperative refraction	Predictive refraction		Refraction error	
		Optic biometry	Acoustic biometry	Optic biometry	Acoustic biometry
RRD group	−1.50 ± 1.72	−1.06 ± 1.40	−0.68 ± 1.51	−0.44 ± 0.77	−0.81 ± 0.75
Control group	−0.39 ± 1.12	−0.67 ± 0.90	−0.18 ± 0.86	0.27 ± 0.71	−0.21 ± 0.64

*Note*. Data are expressed as mean ± standard deviation. RRD, rhegmatogenous retinal detachment.

**Table 3:** Comparison of predicted refraction error measured by acoustic biometry and optic biometry.

	Refractive error (optic biometry)	Refractive error (acoustic biometry)	*P* value	Difference	95% CI
RRD group	−0.44 ± 0.77	−0.81 ± 0.75	*P* < 0.0001	0.37	0.22–0.51
Control group	0.27 ± 0.71	−0.21 ± 0.64	*P* < 0.0001	0.48	0.32–0.65

*Note*. Data are expressed as mean ± standard deviation. The values for refractive error were analyzed using the paired *t*-test. CI, confidence interval; RRD, rhegmatogenous retinal detachment.

**Table 4:** Comparison of predicted refraction error measured by acoustic biometry and optic biometry between the RRD and control groups.

	RRD group	Control group	*P* value	Difference	95% CI
Refractive error (optic biometry)	−0.44 ± 0.77	0.27 ± 0.71	*P* < 0.001	−0.71	−0.41 to −1.01
Refractive error (acoustic biometry)	−0.81 ± 0.75	−0.21 ± 0.64	*P* < 0.001	−0.60	−0.32 to −0.88

*Note*. Data are expressed as mean ± standard deviation. The values for refractive error were analyzed using Student's *t*-test. CI, confidence interval; RRD, rhegmatogenous retinal detachment.

## Data Availability

The data in this paper are available from the corresponding author upon reasonable request.

## References

[1] West S. (1995). Progression of nuclear sclerosis and long-term visual results of vitrectomy with transforming growth factor beta-2 for macular holes. *American Journal of Ophthalmology*.

[2] Koenig S. B., Mieler W. F., Han D. P., Abrams G. W. (1992). Combined phacoemulsification, pars plana vitrectomy, and posterior chamber intraocular lens insertion. *Archives of Ophthalmology*.

[3] Chung T.-Y., Chung H., Lee J. H. (2002). Combined surgery and sequential surgery comprising phacoemulsification, pars plana vitrectomy, and intraocular lens implantation. *Journal of Cataract and Refractive Surgery*.

[4] Savastano A., Savastano M. C., Barca F., Petrarchini F., Mariotti C., Rizzo S. (2014). Combining cataract surgery with 25-gauge high-speed pars plana vitrectomy. *Ophthalmology*.

[5] Caiado R. R., Magalhães O., Badaró E. (2015). Effect of lens status in the surgical success of 23-gauge primary vitrectomy for the management of rhegmatogenous retinal detachment. *Retina*.

[6] Kovacs I., Ferencz M., Nemes J., Somfai G., Salacz G., Recsan Z. (2007). Intraocular lens power calculation for combined cataract surgery, vitrectomy and peeling of epiretinal membranes for macular oedema. *Acta Ophthalmologica Scandinavica*.

[7] Falkner-Radler C. I., Benesch T., Binder S. (2008). Accuracy of preoperative biometry in vitrectomy combined with cataract surgery for patients with epiretinal membranes and macular holes. *Journal of Cataract and Refractive Surgery*.

[8] Manvikar S. R., Allen D., Steel D. H. W. (2009). Optical biometry in combined phacovitrectomy. *Journal of Cataract and Refractive Surgery*.

[9] Hötte G. J., de Bruyn D. P., de Hoog J. (2018). Post-operative refractive prediction error after phacovitrectomy: a retrospective study. *Ophthalmology and Therapy*.

[10] Pongsachareonnont P., Tangjanyatam S. (2018). Accuracy of axial length measurements obtained by optical biometry and acoustic biometry in rhegmatogenous retinal detachment: a prospective study. *Clinical Ophthalmology*.

[11] Jee D., Park Y. R., Jung K. I., Kim E., La T. Y. (2015). Refractive errors in high myopic eyes after phacovitrectomy for macular hole. *International Journal of Ophthalmology*.

[12] Eleftheriadis H. (2003). IOLMaster biometry: refractive results of 100 consecutive cases. *British Journal of Ophthalmology*.

[13] Retzlaff J. A., Sanders D. R., Kraff M. C. (1990). Development of the SRK/T intraocular lens implant power calculation formula. *Journal of Cataract and Refractive Surgery*.

[14] Rose L. T., Moshegov C. N. (2003). Comparison of the Zeiss IOLMaster and applanation A-scan ultrasound: biometry for intraocular lens calculation. *Clinical and Experimental Ophthalmology*.

[15] Kojima T., Tamaoki A., Yoshida N., Kaga T., Suto C., Ichikawa K. (2010). Evaluation of axial length measurement of the eye using partial coherence interferometry and ultrasound in cases of macular disease. *Ophthalmology*.

[16] Byrne S., Ng J., Hildreth A., Danjoux J. P., Steel D. H. (2008). Refractive change following pseudophakic vitrectomy. *BMC Ophthalmology*.

[17] Gao Q., Chen X., Ge J. (2009). Refractive shifts in four selected artificial vitreous substitutes based on Gullstrand-Emsley and Liou-Brennan schematic eyes. *Investigative Opthalmology and Visual Science*.

[18] Mehdizadeh M., Nowroozzadeh M. H. (2009). Postoperative induced myopia in patients with combined vitrectomy and cataract surgery. *Journal of Cataract and Refractive Surgery*.

[19] Iwase T., Sugiyama K. (2006). Investigation of the stability of one-piece acrylic intraocular lenses in cataract surgery and in combined vitrectomy surgery. *British Journal of Ophthalmology*.

[20] Patel D., Rahman R., Kumarasamy M. (2007). Accuracy of intraocular lens power estimation in eyes having phacovitrectomy for macular holes. *Journal of Cataract & Refractive Surgery*.

[21] Petternel V., Menapace R., Findl O. (2004). Effect of optic edge design and haptic angulation on postoperative intraocular lens position change. *Journal of Cataract and Refractive Surgery*.

[22] Olsen T. (2007). Calculation of intraocular lens power: a review. *Acta Ophthalmologica Scandinavica*.

[23] Jeoung J. W., Chung H., Yu H. G. (2007). Factors influencing refractive outcomes after combined phacoemulsification and pars plana vitrectomy. *Journal of Cataract and Refractive Surgery*.

[24] Kim M., Kim H. E., Lee D. H., Koh H. J., Lee S. C., Kim S. S. (2015). Intraocular lens power estimation in combined phacoemulsification and pars plana vitrectomy in eyes with epiretinal membranes: a case-control study. *Yonsei Medical Journal*.

